# The psoas muscle density as a predictor of postoperative complications in elderly patients undergoing rectal cancer resection

**DOI:** 10.3389/fonc.2023.1189324

**Published:** 2023-09-14

**Authors:** Yun-Zhou Xiao, Xiao-Ting Wen, Ying-Ying Ying, Xiao-Yan Zhang, Lu-Yao Li, Zhong-Chu Wang, Miao-Guang Su, Xiang-Wu Zheng, Shou-Liang Miao

**Affiliations:** ^1^ Department of Radiology, PingYang Affiliated Hospital of Wenzhou Medical University, Wenzhou, Zhejiang, China; ^2^ Department of Obstetrics, PingYang Affiliated Hospital of Wenzhou Medical University, Wenzhou, Zhejiang, China; ^3^ Department of Radiology, The First Affiliated Hospital of Wenzhou Medical University, Wenzhou, Zhejiang, China

**Keywords:** rectal cancer, postoperative complications, elderly patient, psoas density, sarcopenia

## Abstract

**Background:**

Muscle depletion that impairs normal physiological function in elderly patients leads to poor prognosis. This study aimed to evaluate the association between total abdominal muscle area (TAMA), total psoas area (TPA), psoas muscle density (PMD), and short-term postoperative complications in elderly patients with rectal cancer.

**Methods:**

All elderly patients underwent rectal cancer resection with perioperative abdominal computed tomography (CT). Complications were assessed according to the Clavien-Dindo classification. Severe complications were defined as grade III-V following the Clavien-Dindo classification. Univariate and multivariate analyses were performed to evaluate risk factors of short-term severe postoperative complications.

**Results:**

The cohort consisted of 191 patients with a mean age of 73.60 ± 8.81 years. Among them, 138 (72.25%) patients had Clavien-Dindo 0- II, 53 (27.75%) patients had severe postoperative complications (Clavien-Dindo III-V), and 1(0.52%) patient died within 30 days of surgery. PMD was significantly higher in the Clavien-Dindo 0-II cohort compared to the Clavien-Dindo III-V cohort (*p*=0.004). Nevertheless, TAMA and TPA failed to exhibit significant differences. Moreover, the multivariate regression analysis implied that advanced age [OR 1.07 95%CI (1.02–1.13) *p*=0.013], male [OR 5.03 95%CI (1.76-14.41) *p*=0.003], high charlson comorbidity index (CCI) score [OR 3.60 95%CI (1.44-9.00) *p*=0.006], and low PMD [OR 0.94 95%CI (0.88-0.99) *p*=0.04] were independent risk factors of Clavien-Dindo III-V.

**Conclusion:**

Preoperative assessment of the PMD on CT can be a simple and practical method for identifying elderly patients with rectal cancer at risk for severe postoperative complications.

## Introduction

Rectal cancer is one of the most common cancers worldwide and a major disease of the elderly. According to the GLOBOCAN project of the WHO cancer research center, the number of new cases of colorectal cancer worldwide in 2018 was about 1.8 million, with approximately 880000 deaths (1). Surgery is the cornerstone of curative therapy for patients with rectal cancer (2). However, the patient will become more vulnerable to postoperative complications since incidence and comorbidity rise steeply with age. Therefore, the prospective identification of patients with an increased risk for postoperative complications could optimize outcomes and guide the therapeutic protocol.

With aging, there are gradual changes in body composition and progressive, as well as systematic loss of skeletal muscle mass. The assessment of skeletal muscle on a single CT slice at the level of the third lumbar vertebra (L3) is strongly correlated with the volume of skeletal muscle in the entire body (3, 4), which can reflect sarcopenia. Numerous studies have revealed that sarcopenia is associated with poor prognosis in various malignancies, comprising gastric cancer (5), colorectal cancer (6), vascular (7), acute mesenteric ischemia (8), and emergency laparotomy surgery (9). Similarly, sarcopenia is commonly observed in elderly patients with rectal cancer, while previous studies have presented conflicting data on its prognostic role in rectal cancer. Chai et al. (10) revealed that sarcopenia defined by total abdominal muscle area (TAMA) was significantly associated with complications. Benedek et al. (11) discovered that total psoas area (TPA) was related to postoperative complications instead of psoas muscle density (PMD), consistent with Wu et al. (9). However, Pekařová et al. (12) and Cuijpers et al. (13) unveiled that PMD was correlated with postoperative complications rather than TPA. Although the indicators of sarcopenia are controversial, the number of cases in our study can tackle this difficulty.

Additionally, there are few studies on the relationship between TAMA, TPA, PMD, and postoperative complications in elderly patients with rectal cancer. Accordingly, this study aimed to investigate the relationship between TAMA, TPA, PMD, and short-term postoperative complications in elderly patients with rectal cancer.

## Materials and methods

### Patient selection

This retrospective study was approved by the ethics committee of PingYang Affiliated Hospital of Wenzhou Medical University. The requirement of patient informed consent was waived owing to the retrospective nature of the study. Our research team reviewed the medical records of 230 consecutive patients with rectal cancer who underwent curative or palliative surgeries from June 2012 to July 2022. Inclusion criteria were patients who were 60 years old or above and had abdominal non-contrast CT scans before surgery. Exclusion criteria were patients who underwent emergency surgeries and had CT scans or clinical data not available, corrupt, or incomplete. Finally, a total of 191 patients were included in our study.

### Radiological data

**Figure 1:**
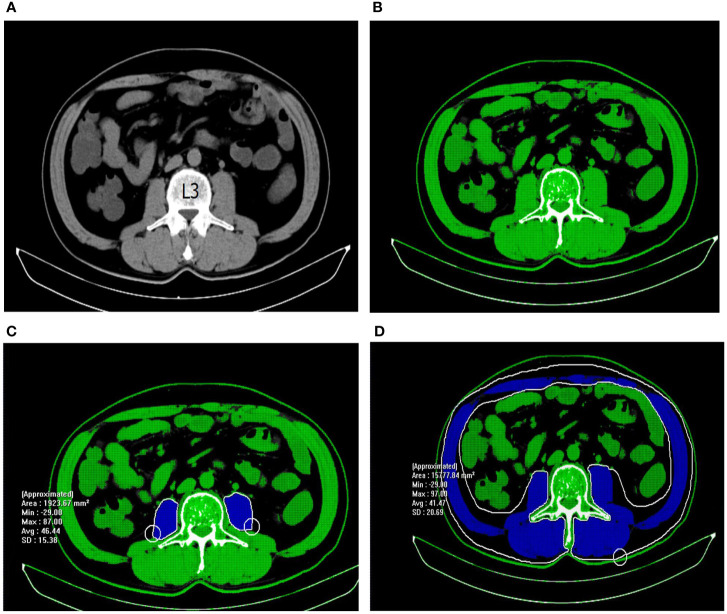
Measurement of abdominal muscle density and area with abdominal non-contrast CT images. **(A)** The third lumbar vertebra was chosen as a landmark. **(B)** Tissues with HU thresholds of − 29 to 150 are automatically outlined in green. **(C)** The selected region of psoas muscle was manually outlined in blue. PMD = 46.44 HU; PMA = 1923.67 mm^2^. **(D)** The selected region of total abdominal muscle was manually outlined in blue. TAMA=15777.84 mm^2^.

Patients underwent abdominal CT scans before the operation, and the CT films were stored in the picture archiving and communication system (PACS) automatically. Total abdominal muscle area (TAMA), psoas muscle area (PMA), density (PMD), and visceral fat area (VFA) were measured at PACS using preoperative abdominal non-contrast CT images by professional imaging software (INFINITT PACS software version 3.0.11.3 BN17 32 bit, INFINITT Healthcare Co., Ltd, Seoul, Korea). As illuminated in **Figure 1**, TAMA, TPA, PMD, and VFA were assessed at the cross sections in the third lumbar vertebra (L3) level where both transverse processes were visible. Predefined Hounsfield unit (HU) thresholds were employed for specific tissue demarcation. The Hounsfield unit threshold ranges of -29 – 150 and -150 – -50 were identified as skeletal muscle and visceral fat, respectively. Tissue boundaries were outlined manually as needed.

TAMA is composed of the psoas, quadratus lumborum, erector spinae, external and internal obliques, transversus, and rectus abdominis. PMA was measured by summing the left and right psoas muscle areas. PMD was quantified as the mean muscle attenuation for the cross-sectional psoas muscle. TAMA and TPA were normalized for height (m^2^) and reported as L3 skeletal muscle index (cm^2^/m^2^) and psoas muscle index (cm^2^/m^2^), respectively. According to the cutoff levels of VFA for metabolic syndrome in the Japanese population (14), visceral obesity was defined as men with a VFA > 130 cm^2^ and women with a VFA > 90 cm^2^. CT scans were executed by a single operator, skilled in radiological anatomy and body composition analysis, and blinded to the patient outcomes to avoid bias and possible inter-observer variation.

### Data collection

Preoperative patient details and postoperative outcome data were retrieved from electronic patient records. The following data were collected and analyzed retrospectively (1): the patient demographic and clinicopathological features, including age, gender, body mass index (BMI), hemoglobin concentration (a hemoglobin concentration < 120 g/L in men and < 110 g/L in women was defined as anemia), plasma albumin concentration (a plasma albumin concentration < 35g/L was defined as hypoalbuminemia), white blood cell (WBC) count, charlson comorbidity index (CCI) score (15), American Society of Anesthesiology (ASA) grade, nutritional risk screening 2002 (NRS 2002) scores, previous abdominal surgery, previous radiotherapy and chemotherapy, tumor location, histologic type, tumor size, and tumor node metastasis (TNM) stage of tumor (2); operative details, consisting of epidural anesthesia, laparoscopy-assisted operation, combined resection, surgical duration, estimated blood loss, and operation mode (3); postoperative short-term outcomes, composed of postoperative complications within 30 days after surgery. According to the Clavien-Dindo classification, complications were divided into Clavien-Dindo 0-II and Clavien-Dindo III-V, among which Clavien-Dindo III-V complications were defined to be significant (16).

### Statistical analysis

Normally distributed continuous data were presented as mean ± SD, and the differences between groups were compared using Student’s t-test. Non-normally distributed variables were presented as median with interquartile range (IQR), and the Mann-Whitney U-test was used. Categorical data were compared using the chi-squared test or Fisher’s exact probability test. Variables with a value of *P* < 0.2 in the univariate analyses were included in the subsequent multivariate forward logistic regression analysis. Results are represented as odds ratio (OR) [95% confidence interval]. All tests were 2-sided, and a P-value <0.05 indicated statistical significance. The statistical analyses were performed using the SPSS statistics version 25.0 (IBM, Armonk, NY, USA) software programs.

## Results

### Patient demographics

A total of 191 patients satisfying our inclusion criteria were included in our analysis. The baseline characteristics are detailed in **Table 1**. The tumor location was mostly located in the upper (115, 60.21%), and 168 (87.96%) patients underwent Dixon surgery. Among them, 88 (46.07%) resections were performed via laparotomy. Most patients had an ASA score ≤ 2 (141, 73.82%), an NRS2002 score < 3 (172, 90.05%) was the most common, and only 75 (39.27%) patients presented a CCI score of 0. There were 82 (42.93%) patients with anemia and 28 (14.66%) patients with hypoalbuminemia.

**Table 1:** Patient Demographic and Clinical Characteristics.

Clavien-Dindo Classification				
	Total (N=191)	0-II (N=138)	III-IV (N=53)	*P*-value
**TAMA,** median (0.25,0.75),cm^2^/m^2^	40.71 (33.90,46.75)	41.17 (34.22,47.61)	40.39 (31.80,45.15)	0.074
**TPA,** median (0.25,0.75),cm^2^/m^2^	4.75 (3.77,5.72)	4.74 (3.77,5.80)	4.83 (3.87,5.52)	0.504
**PMD,** median (0.25,0.75),**HU**	38.74 (32.95,42.26)	39.21 (33.95,43.08)	36.69 (30.45,40.46)	0.004 **
**Surgical duration,** median (0.25,0.75),**min**	175 (141.5,210)	166.5 (140,204.25)	190 (150,225)	0.087
**BMI,** median (0.25,0.75),**kg/m^2^**	22.04 (19.98,24.22)	22.04 (19.79,24.22)	22.22 (20.20,23.88)	0.947
Age, median (0.25,0.75),yeas	74(66,81)	70.5(65,78)	80(72,85)	0.000 **
Gender				0.169
Female, n (%)	65 (34.0%)	51 (37.0%)	14 (26.4%)	
Male, n (%)	126 (66.0%)	87 (63.0%)	39 (73.6%)	
Tumor location				0.382
Upper, n (%)	115 (60.2%)	83 (60.1%)	32 (60.4%)	
Lower, n (%)	27 (14.1%)	21 (15.2%)	6 (11.3%)	
Middle, n (%)	48 (25.1%)	34 (24.6%)	14 (26.4%)	
Whole, n (%)	1 (0.5%)	0 (0.0%)	1 (1.9%)	
TNM stage				0.553
I, n (%)	39 (20.4%)	30 (21.7%)	9 (17.0%)	
II, n (%)	75 (39.3%)	53 (38.4%)	22 (41.5%)	
III, n (%)	68 (35.6%)	47 (34.1%)	21 (39.6%)	
IV, n (%)	9 (4.7%)	8 (5.8%)	1 (1.9%)	
Histologic type				0.784
Differentiated ^a^, n (%)	173 (90.6%)	124 (89.9%)	49 (92.5%)	
Undifferentiated ^b^, n (%)	18 (9.4%)	14 (10.1%)	4 (7.5%)	
ASA **score**				0.000 **
≤2, n (%)	141 (73.8%)	112 (81.2%)	29 (54.7%)	
>2, n (%)	50 (26.2%)	26 (18.8%)	24 (45.3%)	
NRS2002 **score**				0.044 *
≥3, n (%)	19 (9.9%)	10 (7.2%)	9 (17.0%)	
<3, n (%)	172 (90.1%)	128 (92.8%)	44 (83.0%)	
Visceral obesity, n (%)	92 (48.2%)	65 (47.1%)	27 (50.9%)	0.634
CCI **score**				0.000 **
0, n (%)	75 (39.3%)	67 (48.6%)	8 (15.1%)	
≥1, n (%)	116 (60.7%)	71 (51.4%)	45 (84.9%)	
**Previous radiotherapy and chemotherapy**, n (%)	5 (2.6%)	4 (2.9%)	1 (1.9%)	1.000
Previous abdominal surgery, n (%)	27 (14.1%)	20 (14.5%)	7 (13.2%)	0.819
Epidural anesthesia, n (%)	37 (19.4%)	28 (20.3%)	9 (17.0%)	0.604
Laparoscopy-assisted operation, n (%)	103 (53.9%)	71 (51.4%)	32 (60.4%)	0.268
Operation mode,				0.907
Dixon, n (%)	168 (88.0%)	121 (87.7%)	47 (88.7%)	
Hartmann, n (%)	1 (0.5%)	1 (0.7%)	0 (0.0%)	
ISR, n (%)	5 (2.6%)	4 (2.9%)	1 (1.9%)	
Miles, n (%)	16 (8.4%)	11 (8.0%)	5 (9.4%)	
TAE, n (%)	1 (0.5%)	1 (0.7%)	0 (0.0%)	
Combined resection, n (%)	29 (15.2%)	20 (14.5%)	9 (17.0%)	0.668
Intraoperative bleeding >300 ml, n (%)	24 (12.6%)	17 (12.3%)	7 (13.2%)	0.868
Tumor size, cm				0.653
≥4, n (%)	114 (59.7%)	81 (58.7%)	33 (62.3%)	
<4, n (%)	77 (40.3%)	57 (41.3%)	20 (37.7%)	
WBC > 10 G/L, n (%)	20 (10.5%)	13 (9.4%)	7 (13.2%)	0.444
Hypoalbuminemia, n (%)	28 (14.7%)	15 (10.9%)	13 (24.5%)	0.017 *
CEA **> 9.7 ng/mL**, n (%)	60 (31.4%)	44 (31.9%)	16 (30.2%)	0.821
Anemia, n (%)	82 (42.9%)	60 (43.5%)	22 (41.5%)	0.806
CA199 **> 37 U/mL**, n (%)	25 (13.1%)	20 (14.5%)	5 (9.4%)	0.353

*p<0.05, ** p<0.01.

TAMA, Total Abdominal Muscle Area; TPA, Total Psoas Area; PMD, Psoas Muscle Density; BMI, body mass index; TNM, Tumor Node Metastasis; ASA, American Society of Anesthesiology; NRS2002, Nutritional Risk Screening 2002; CCI, Charlson Comorbidity Index; WBC, White Blood Cell; CEA, Carcino Embryonic Antigen;CA199, Cancer Antigen 199.

a Undifferentiated carcinomas include poorly differentiated adenocarcinomas, signet ring cell carcinomas, and mucinous carcinomas.

b Differentiated carcinomas include well or moderately differentiated, tubular or papillary adenocarcinomas.

### Group comparison and multivariate analysis

According to the Clavien-Dindo classification, 138 (72.25%) and 53 (27.75%) had Clavien-Dindo 0-II and severe postoperative complications (Clavien-Dindo III-V), respectively, and 1(0.52%) patient died within 30 days of surgery. There was significant association in advanced age (*p*<0.01), high ASA score (*p*<0.01), high CCI score (*p*<0.01), high NRS2002 score (*p*=0.044), PMD (*p*=0.004), and hypoalbuminemia (*p*=0.017) between Clavien-Dindo 0-II and Clavien-Dindo III-V. TAMA (*p*=0.123), TPA (*p*=0.543), and other factors were not significantly associated between the two groups.

The results of the multivariate analysis of factors associated with severe postoperative complications (grade III-V) are shown in **Table 2**.The multivariate regression analysis suggested that advanced age [OR 1.07 95%CI (1.02-1.13) *p*=0.013], male [OR 5.03 95%CI (1.76-14.41) *p*=0.003], high CCI score [OR 3.60 95%CI (1.44-9.00) *p*=0.006], and low PMD [OR 0.94 95%CI (0.88-0.99) *p*=0.04] were independent risk factors of Clavien-Dindo III-V.

**Table 2:** Multivariate logistic regression analyses of factors influencing Clavien-Dindo III-IV in rectal cancer resection.

	*P*-value	OR-value	OR 95%CI
**ASA score≤2**	0.263	1.62	0.70-3.76
**CCI score≥1**	0.006	3.60	1.44-9.00
**NRS2002 score≥3**	0.71	1.25	0.39-4.00
**PMD**	0.04	0.94	0.88-0.99
**TAMA**	0.079	0.954	0.91-1.01
**Age**	0.013	1.07	1.02-1.13
**Male**	0.003	5.03	1.76-14.41
**Surgical duration**	0.07	1.01	1.00-1.01
**Hypoalbuminemia**	0.557	1.32	0.50-3.46

*p<0.05, ** p<0.01.

ASA, American Society of Anesthesiology; CCI, Charlson Comorbidity Index; NRS2002, Nutritional Risk Screening 2002; PMD, Psoas Muscle Density; TAMA, Total Abdominal Muscle Area.

## Discussion

The main finding of the present study was that low PMD, but not TAMA and TPA, is an independent risk factor for severe postoperative complications in elderly patients with rectal cancer. To our knowledge, this is the first study to demonstrate the utility of low PMD based on CT for identifying elderly patients with rectal cancer at risk for postoperative complications.

Low muscle density is an indicator of intramuscular fat deposition (17) and is correlated with physical performance (18). It could reflect the decline in muscle quality. A growing body of evidence revealed that low muscle mass was associated with postoperative complications in various surgical procedures. Moreover, low muscle area (TAMA, TPA) was related to severe postoperative complications in colorectal cancer (19, 20). Similarly, Herrod et al. (21) discovered that psoas density was highly predictive of postoperative morbidity in colorectal cancer patients of all ages. Additionally, Margadant et al. (22) unveiled that low psoas density was ascribed to major postoperative complications in elderly patients who underwent surgery for colorectal cancer. Colorectal cancer develops from the colon or rectum. However, colon cancer and rectal cancer are two separate tumor entities requiring distinct treatment approaches (23, 24) since they have different molecular developmental mechanisms and metastatic patterns (25, 26). Therefore, colon cancer and rectal cancer should be considered independently in evaluating postoperative complications. Our study emphasized rectal cancer, especially among the elderly. Elderly people represent almost all patients diagnosed with and treated for rectal cancer. This trend is likely to become more apparent in the future. Surgical management and treatment decisions for this disease become increasingly complex, while only a few reports specifically wrestle from older patients (2). Frailty is crucial in cancer. The incidence of frailty in elderly patients with cancer is particularly high (27). Frailty is a state of extreme vulnerability to stressors, leading to adverse health outcomes (28–30). Muscle tissue assessment is a quick and easy way to quantify a patient’s level of frailty (31). Age is an independent risk factor for decreased muscle quality (32). This was one of the main reasons to emphasize elderly patients in this study. Additionally, our study also verified that advanced age is an independent risk factor for severe postoperative complications of elderly rectal cancer. With increasing age, various physiological systems decline in the human body, forming a complex, multidimensional, and periodic state of physiological reserve reduction. As a result, resilience and adaptability decreased, and vulnerability to stressors increased (32–35). Consequently, our study lays a little foundation for the preoperative preparation of the elderly for rectal cancer.

Interestingly, our findings suggested that TAMA and TPA were not associated with the short-term postoperative complications of elderly rectal cancer, inconsistent with the studies of Uehara et al. (36) and Jochum et al. (37). Psoas muscle density, instead of area, was associated with the short-term postoperative complications of elderly rectal cancer. Its possible reasons are described as follows. Firstly, intramuscular fat infiltration does not affect its area, implying that PMD decreases while TAMA and TPA remain unchanged. It was assumed in our study that muscle quality rather than quantity was more critical in the estimation of complications risk. Secondly, it can be speculated that muscle fat infiltration can lead to a decrease in muscle strength, TAMA and TPA reflect muscle mass, and PMD reflects muscle strength. Moreover, Zhang et al. (38) uncovered that muscle strength is a better predictor of postoperative complications and overall survival of gastric cancer compared with muscle mass. Similarly, low muscle density, but not area (TAMA, TPA), is an independent risk factor for severe postoperative complications of rectal cancer (39).

Our study also revealed that high CCI scores and males were independent risk factors for severe postoperative complications of elderly rectal cancer, consistent with some previous studies (12, 36, 39). Increasing age is considered a decisive risk factor in association with concomitant diseases (40). It may induce poorer surgical outcomes (41, 42). Portale et al. (43) demonstrated that higher CCI was an independent predictor of short-term results of patients who underwent laparoscopic curative resection for rectal cancer, in line with our study. Research of 196 patients who underwent rectal cancer resection suggested that male patients were an independent risk factor for severe postoperative complications (44). The male patients influence the postoperative complications since the male pelvis is technically more challenging (45).

The present study had several limitations. First, it was a retrospective, single-institution analysis. Second, potential bias and certain limitations with respect to their internal validity and generalization exist due to the limited number of patients in our study. Third, the association of PMD with postoperative complications according to age stratification was not evaluated although we concluded that age had an effect on PMD and postoperative complications. In the future, further studies with a larger study population should be conducted to verify the relationship between PMD and postoperative complications according to age stratification. Finally, the long-term outcome was not analyzed in this study.

## Conclusions

To summarize, this study demonstrated that preoperative assessment of the PMD on CT can be a simple and practical method for identifying elderly patients with rectal cancer at risk for severe postoperative complications. PMD should be employed to augment existing methods of patient-risk stratification before surgery.

## Data availability statement

The raw data supporting the conclusions of this article will be made available by the authors, without undue reservation.

## Ethics statement

The studies involving human participants were reviewed and approved by Ethics Committee of Pingyang County People’s Hospital. Written informed consent for participation was not required for this study in accordance with the national legislation and the institutional requirements. Written informed consent was obtained from the individual(s) for the publication of any potentially identifiable images or data included in this article.

## Author contributions

Y-ZX, S-LM, X-TW, and X-WZ conceived and designed the experiments and were responsible for data analysis and writing the manuscript. Y-YY, L-YL, X-YZ, and Z-CW were responsible for providing the clinical samples. M-GS was responsible for data collection. All authors contributed to the article and approved the submitted version.
